# Suprachoroidal injection of triamcinolone acetonide as adjuvant to surgical treatment of epiretinal membrane

**DOI:** 10.1186/s40942-024-00623-8

**Published:** 2025-01-09

**Authors:** Francesco Morescalchi, Federico Gandolfo, Vito Romano, Andrea Baldi, Francesco Semeraro

**Affiliations:** 1 Eye Clinic, Department of Medical and Surgical Specialties, Radiological Sciences and Public Health, University of Brescia, 25121 Brescia, Italy; 2 ASST Spedali Civili di Brescia: Azienda Socio Sanitaria Territoriale Degli Spedali Civili di Brescia, 25123 Brescia, Italy

**Keywords:** Epiretinal membrane, Suprachoroidal injection, Triamcinolone acetonide, Vitrectomy

## Abstract

**Background:**

To analyse the effect of suprachoroidal injection (SChI) of triamcinolone acetonide (TA) on macular thickness (CRT), ectopic inner foveal layer thickness (EIFL-T) and best corrected visual acuity (BCVA) in pseudophakic patients undergoing vitrectomy for epiretinal membrane (iERM) compared to intravitreal injection of TA (IVTA).

**Methods:**

Prospective matched comparison of patients undergoing vitrectomy for Govetto stage 3 and 4 iERM. 25 eyes receiving IVTA (G-1) were compared to 23 eyes receiving SChI-TA (G-2) during vitrectomy. Primary outcome was change in BCVA, CRT, EIFL-T before surgery and 1, 3 and 6 months after surgery. Secondary outcome was the incidence of cystoid macular edema (CME).

**Results:**

Six months after surgery, G2 had a greater mean reduction in CRT (−222 µm vs −131 µm) and EIFL-T (−200 µm vs −104 µm) than G1. BCVA improved more in G2 than in G1 (p = 0.02). Foveal depression reformed in 43% of cases in G-2 and 16% of cases in G-1. Incidence of postoperative CME was 16% in G-1 and 4.3% in G-2.

**Conclusions:**

During vitrectomy for iERM, SChI-TA was more effective than IVTA in reducing CRT and EIFL-T and improving BCVA. SChI-TA was effective in preventing postoperative CME. SChI-TA treatment was safe and reproducible and did not affect postoperative IOP.

*Trial registration* NP6289—June 18th, 2024 (retrospectively registered).

## Background

Idiopathic Epiretinal Membranes (iERMs) occur in almost 6% of the population aged over 60 years and can cause puckering and thickening of the macula, leading to blurred vision and metamorphopsia [1]. When visual symptoms become significant, small gauge pars-plana vitrectomy (PPV) and ERM with or without peeling of the inner limiting membrane (ILM) is indicated. After surgery, the removal of the anteroposterior and tangential traction of the membrane allows the macular distortion to be reduced and helps to resolve the retinal edema. Intravitreal injection of triamcinolone-acetonide (IVTA) at the end of surgery may accelerate the reduction of macular thickening and improve visual acuity more rapidly [2–4]. However, some investigators found no differences in best corrected visual acuity (BCVA) and central retinal thickness (CRT) between those who received IVTA and those who did not [5, 6]. The short duration of TA in vitrectomized eyes may explain the mixed results in the literature [7, 8].

Suprachoroidal injection of triamcinolone acetonide (SChI-TA) is a new method for ocular drug delivery that has several theoretical advantages over the traditional intravitreal injection route [9].

1. The suprachoroidal space (SchS) could serve as a reservoir, releasing a drug for a longer period of time than the intravitreal route.
2. SChI-TA allows a high drug concentration to be achieved in the posterior segment of the eye.
3. SChI-TA avoids direct contact of the drug with the retina, reducing the potential direct toxic effects, including the risk of infection.
4. SChI-TA reduces the risk of increased intraocular pressure, compared to the intravitreal route.

The present study was designed to compare the effects of TA in two different routes of drug administration, namely IVTA and SChI-TA, performed intraoperatively during vitreoretinal surgery in patients with advanced iERM.

## Methods

This prospective, randomized and comparative pilot study was conducted at the University Eye Clinic of the “Spedali Civili di Brescia” (Italy). All study participants provided written informed consent. The hospital's institutional review board approved the study protocol (clinical trial number: NP6289), which was conducted in accordance with the ethical principles of the Declaration of Helsinki.

### Inclusion criteria

Eligible patients were pseudophakic patients between the ages of 50 and 85 years with advanced idiopathic iERM who were referred to our retina service. The time between cataract extraction and PPV was not less than 3 months to exclude inflammatory phenomena due to anterior segment surgery [10]. The diagnosis of iERM was made as follows: ERM with CRT > 300 µm verified by optical coherence tomography (OCT) and classified according to the OCT-based staging system devised by Govetto et al. as stageS-3 and S-4 [11].

Patients with S-1 and S-2 iERM, with ERM secondary to other ocular pathologies (retinal vascular disease, retinal detachment, uveitis, etc.), affected by age related maculopathy, diabetes or glaucoma were excluded.

### Clinical evaluation

The following examinations were performed prior to intraocular procedure and at each visit:

1. Slit lamp anterior inspection and intraocular pressure (IOP) assessment by Goldmann applanation tonometry;
2. BCVA was measured by Snellen Chart and the Snellen Equivalent (SE) values were converted to LogMAR scale;
3. Posterior segment inspection by indirect ophthalmoscopy;
4. CRT and ectopic inner foveal layer thickness (EIFL-T) were measured using a Spectralis HRA-OCT (Heidelberg Engineering-Heidelberg-Germany). The Radial Line Scan or the Raster Scan patterns were used for retinal imaging. The “follow-up” Raster Scan pattern was used to monitor the anatomical changes of the macula after surgery.

On OCT, EIFL appears as a continuous hyporeflective (extension of the inner nuclear layer-INL-) or hyperreflective (extension of the inner plexiform layer) band across the fovea.

EIFL-T was manually measured with the built-in caliper tool of the HEYEX^®^ software. EIFL-T was the distance between the outer border of the INL to the inner edge of the ILM in the subfoveal area. Two expert retinal specialists masked to the participants' clinical details independently evaluated the OCT images. A third masked retina specialist adjudicated the discrepancies.

### Surgical technique

All patients underwent a 25-Gauge (G) PPV performed by two experienced surgeons (FM and FG) under local anaesthesia; at the end of the procedure they were intraoperatively randomized in a 1:1 ratio (using a sequence obtained from www.randomizer.org) to IVTA (Group-1: G-1) or a SChI-TA (Group-2: G-2).

The PPV was performed with the following steps: (1) induction of posterior hyaloid detachment by active aspiration with the vitreous cutter; (2) removal of core and peripheral vitreous gel; (3) complete peeling of both the ERM and ILM within the major vascular arcades, after the intravitreal injection of a vital dye (Twin^™^—AL.CHI.MIA. S.r.l.—Italy); injection of 0.1 mL (4 mg) of preservative free TA (TRIACORT^™^—PharmaTex Italia S.r.l.—Italy).

G-1 received IVTA, through a standard 30 G needle, via one of the 25 G trocars just before sclerotomy closure.

**Fig. 1:**
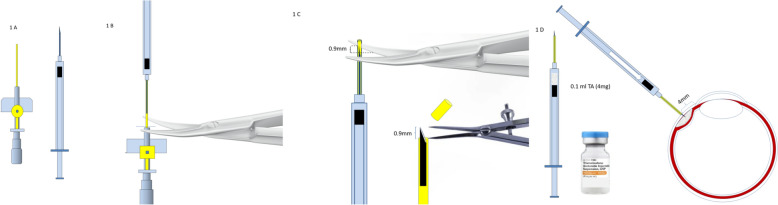
SChI-TA preparation and injection procedure. **A** SChI-TA was performed using a 1 mL insulin syringe equipped with a 29 G needle and a 20 G intravenous cannula (Venflon™ B yellow) with a 25 mm long cannula having an outer diameter of 0.9 mm. **B** The Venflon stylet was removed, the 29G needle was inserted into the lumen of the cannula, and the plastic cannula was cut at the base. **C** The end of the cannula was cut with Westcott scissors, exposing approximately 0.9 mm of the tip of the needle. The cutting was performed under an operating microscope; a caliber was used to verify the length of the exposed needle tip. **D** The insulin syringe was then filled with 0.1 mL of Triamcinolone Acetonide (4 mg). The injection procedure was performed as follows: (1) the intraocular pressure was raised to 25–28 mm Hg; (2) the syringe was held perpendicular to the scleral plane; (3) the needle was inserted 4–5 mm from the limbus in the inferior temporal quadrant, with the blunt side facing posteriorly; (4) after creating a small dimple on the sclera, while maintaining steady and firm pressure on the plunger, the injection of TA was performed steadily and slowly

G-2 received the same dose of drug via the suprachoroidal injection. The SChI-TA was performed using a 29 G needle, and a customized plastic protection exposed the tip of the needle only for the 0.9 mm required to inject the drug in the correct location (Fig. 1).

### Follow-up

All patients were examined after 24 h for any complication like raised IOP or inflammation. Patients were examined after 1 week, 1, 3 and 6 months; complete ophthalmological checks including BCVA, IOP measurement and indirect ophthalmoscopy were performed, and OCT images were acquired again using the “follow-up” raster scan pattern.

### Statistical analysis

Shapiro–Wilk’s test assessed that all data were normally distributed (P > 0.05). Repeated-measures analysis of variance (RMANOVA) with Greenhouse–Geisser and Bonferroni corrections was performed for the identification of significant differences in the changes in BCVA, CRT, and EIFL-T between the G-1 and G-2. Independent-samples t-tests were used to determine the statistical significance of intergroup differences in BCVA, CRT, and EIFL-T. All statistical analyses were performed using SPSS software V.20 (SPSS Chicago-IL-USA). A p-value of < 0.05 was considered statistically significant.

## Results

50 patients (50 eyes) were consecutively enrolled in the study, from June 2022 to March 2023: 25 patients (25 eyes) in G-1, 25 patients (25 eyes) in G-2. In G-1, 25 patients completed 6 months of follow-up. In G-2, 23 patients completed the 6-month follow-up (2 patients lost the 6th month follow-up visit).

In total the results of 48 eyes were considered in the statistical analysis (25 eyes in G-1vs 23 eyes in G-2). No differences were found between the two groups in terms of demographics and clinical characteristics, listed in Table 1. Results are listed in Tables 2, 3 and 4.

**Table 1:** Patient Demographic and Baseline Characteristics

	Group I: IVTA (N = 25)	Group II: SChI-TA (N = 23)	P value
Age: mean (SD), years	68.5 (13)	70.7 (12)	0.53
Sex: n (%)			
Male	10 (40%)	8 (34%)	0.72
Female	15 (60%)	15(65%)	0.72
Visual Acuity: mean (SD), Log MAR	0.45 (± 0.16)	0.52 (± 0.18)	0.80
Snellen equivalent	20/56 (± 20/158)	20/66 (± 20/141)	
Baseline CRT (SD), µm	505(± 69)	543 (± 120)	0.17
Baseline EIFL-T (SD), µm	102 (± 30)	124 (± 71)	0.15
Pseudophakic > 6 months	23 (92%)	20 (86%)	0.75
Pseudophakic since 6–3 months	2 (8%)	3 (13%)	0.75
Stage 3 ERM	18 (72%)	16 (69%)	0.90
Stage 4 ERM	7 (28%)	7 (30%)	0.90
Presence of macular pseudocysts	2 (8%)	2 (8, 6%)	0.97
Presence of parafoveal retinoschisis	2 (8%)	2 (8, 6%)	0.97
IOP mmHg	16.6 (± 1.9)	16.9 (± 2.23)	0.90

*CRT* central retinal thickness, *EIFL-T* Ectopic Inner Foveal Layer Thickness, *IOP* Intra Ocular Pressure, *SD* standard deviation, Group I = vitrectomy with Intra Vitreal Injection of Triamcinolone Acetonide (IVTA); Group II = vitrectomy with Supra Choroideal Injection of Triamcinolone Acetonide (SChI-TA)

**Table 2:** Changes in Visual Acuity, Central Retinal Thickness, EIFL-Thickness and Intra Ocular Pressure over 6 Months after Surgery in stage 3–4 iERM

	Baseline	1 Month	3 Months	6 Months
BCVA				
G-1 LogMAR ± SD	0.45 ± 0.16	0.32 ± 0.2 (*)	0.23 ± 0.12(*)	0.23 ± 0.11 (*)
(Snellen eq. ± SD)	(20/56 ± 20/158)	(20/41 ± 20/100)	(20/33 ± 20/141)	(20/33 ± 20/126)
G-2 LogMAR ± SD	0.52 ± 0.18	0.25 ± 0.13 (*)	0.16 ± 0.11 (*)	0.15 ± 0.13 (*)
(Snellen eq. ± SD)	(20/66 ± 20/141)	(20/35 ± 20/89)	(20/28 ± 20/126)	(20/28 ± 20/120)
p	0.85	0.7	**0.03**	**0.02**
CRT				
G-1	505 ± 69 µm	456 ± 103 (*) µm	400 ± 72 (*) µm	374 ± 82(*) µm
G-2	543 ± 120 µm	404 ± 98 (*) µm	351 ± 82 (*) µm	320 ± 77 (*) µm
p	0.17	0.08	**0.03**	**0.02**
EIFL-T				
G-1	217 ± 70 µm	178 ± 60 (*) µm	132 ± 54 (*) µm	113 ± 54 (*) µm
G-2	272 ± 115 µm	148 ± 38 (*) µm	82.6 ± 55 (*) µm	72 ± 45 (*) µm
p	0.056	**0.046**	**0.002**	**0.001**
IOP				
G-1 mmHg	16.6 ± 1.1	17.8 ± 2.99	17.6 ± 2.29	16.9 ± 0.9
G-2 mmHg	16.7 ± 1.4	18.6 ± 2	17 ± 1.96	17.2 ± 1.27
p	0.78	0.28	0.33	0.34

Bold indicated statistically significant difference

(*)P < 0.05 compared with baseline values for the same group. P values presented are for the difference between the 2 groups at baseline and at 1, 3 and 6 months

G-1 = vitrectomy with Intra Vitreal Injection of Triamcinolone Acetonide. G-2 = vitrectomy with Supra Choroideal Injection of Triamcinolone Acetonide. *BCVA* Best Corrected Visual Acuity, *CRT* central retinal thickness, *EIFL-T* Ectopic Inner Foveal Layer Thickness, *IOP* Intra Ocular Pressure

**Table 3:** Mean reduction in CRT and EIFL-T in both groups

Reduction from baseline		1 Month	3 Months	6 Months
G-1 CRT	Reduction in µm %	−49 ± 76 µm (−**9.7**% ± 10)	−105 ± 62 µm (−**20.4**% ± 11)	−131 ± 56 µm (−**26.4**% ± 10)
G-2 CRT	Reduction in µm%	−139 ± 101 (−**25**% ± 17)	−192 ± 138 (−**33,2**% ± 20)	−222 ± 132 (−**39,1**% ± 18)
p		**0.001**	**0.006**	**0.003**
G-1 EIFL-T	Reduction in µm%	−39 ± 76 µm (−**17.9**% ± 20)	−84 ± 70 µm (−**36.2**% ± 27)	−104 ± 69 µm (−**46.6**% ± 23)
G-2 EIFL-T	Reduction in µm%	−124 ± 91 (−**45**% ± 21)	−189 ± 110 (−**65**% ± 26)	−200 ± 110 (−**68**% ± 25)
p		** < 0.0001**	** < 0.0001**	** < 0.0001**

Bold indicated statistically significant difference

The magnitude of reduction in CRT and EIFL-T was statistically significant at each follow-up visit

G-1 = vitrectomy with Intra Vitreal Injection of Triamcinolone Acetonide, G-2 = vitrectomy with Supra Choroideal Injection of Triamcinolone Acetonide, *CRT* central retinal thickness, *EIFL-T* Ectopic Inner Foveal Layer Thickness

**Table 4:** Evolution of EIFL-T in Govetto Stage-4 patients

EIFL-T (mean ± SD), µm	Baseline	1 Month	3 Months	6 Months
G-1 (n.7)	249 ± 59	203 ± 88	144 ± 46	133 ± 29
G-2 (n.7)	327 ± 160	183 ± 111	99 ± 29	84 ± 40
p	0.25	0.7	**0.04**	**0.02**

Bold indicated statistically significant difference

Evolution of EIFL-T in patients with Govetto stage 4 iERM. Starting from the 3rd month of follow-up, G2 had a greater reduction in EIFL-T compared to G1

G-1 = vitrectomy with Intra Vitreal Injection of Triamcinolone Acetonide; G-2 = vitrectomy with Supra Choroideal Injection of Triamcinolone Acetonide; *EIFL-T* Ectopic Inner Foveal Layer Thickness

Mean BCVA improved in G-1 from 0.45-logMAR (SE:20/56 ± 20/158) at baseline to 0.23-LogMAR (SE:20/33 ± 20/132) at 6 months after surgery

Mean BCVA improved in G-2 from 0.52-LogMAR (SE:20/66 ± 20/141) at baseline to 0.15-LogMAR (SE:20/28 ± 20/120) at 6 months after surgery

Mean CRT and EIFL-T in G-1 decreased from 505 µm(± 69) and 217 µm(± 70) before surgery to 374 µm(± 82) and 113 µm(± 54), respectively, six months after surgery

Mean CRT and EIFL-T in G-2 decreased more rapidly and significantly, from 543 µm(± 120) and 272 µm(± 115) before surgery to 320 µm(± 77) and 72 μm(± 45) six months later

In percentage terms, CRT and EIFL-T decreased 26% and 46.6% in G-1 and 39.1% and 68% in G2

There was a statistically significant difference in postoperative BCVA, CRT and EIFL-T between the two groups in favour of G-2 after 3 and 6 months (p < 0.05)

**Fig. 2:**
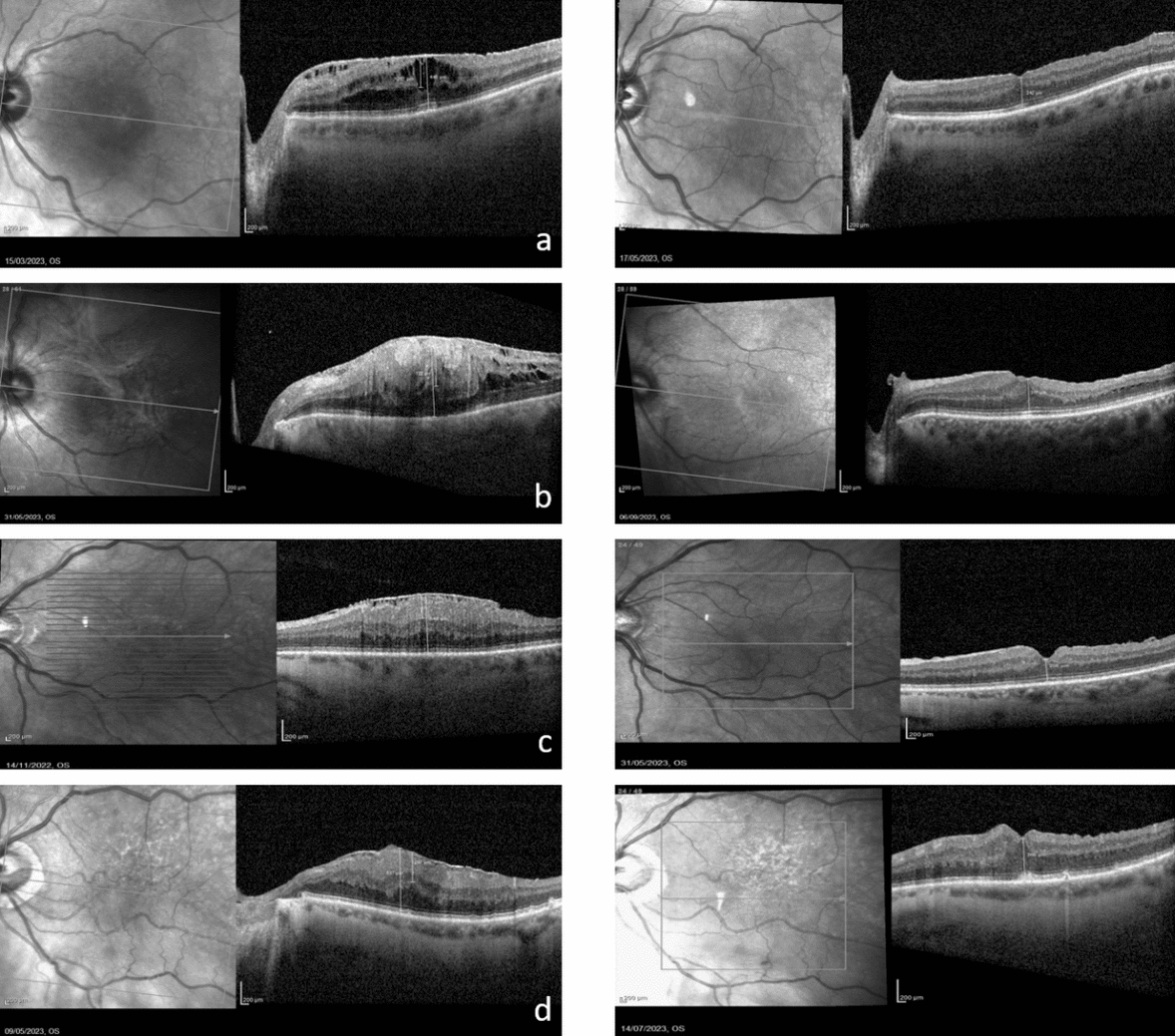
This figure shows the OCT images of 4 patients before (left) and after (right) vitrectomy with SChI-TA. **a** 68 year old male patient with S-3 iERM in left eye (LE). CRT: 440 μm; EIFL-T: 257 μm and cystic spaces in the inner layers; BCVA: 20/66. After 2 months CRT decreased to 242 μm, EIFL was no longer visible, slightly reappearance of the foveal depression; BCVA: 20/28. **b** 64 year old female patient with S-4 iERM in LE. CRT: 710 μm; EIFL-T: 418 μm; BCVA: 20/200. After 4 months CRT decreased to 317 μm, EIFL was no longer visible, slightly reappearance of the foveal pit; BCVA: 20/23. **c** 66 year old male patient with S-4 iERM in LE. BCVA 20/100; CRT: 400 μm; EIFL-T of 300 μm. After 6 months CRT decreased to 315 μm; EIFL disappeared, foveal pit reformed; BCVA: 20/30. **d** 84 year old male patient with S-3 ERM in LE. BCVA: 20/66; CRT: 537 μm; EIFL-T: 237 μm. After 2 months, CRT reduced to 366 μm, EIFL no longer visible, slightly reappearance of the foveal depression; BCVA: 20/24. *SChI-TA* suprachoroidal injection of triamcinolone acetonide, *S* Govetto Stage, *iERM* idiopathic epiretinal membrane, *LE/RE* left/right eye, *CRT* central retinal thickness, *EIFL-T* ectopic inner foveal layer thickness, *BCVA* best corrected visual acuity in Snellen equivalent

**Fig. 3:**
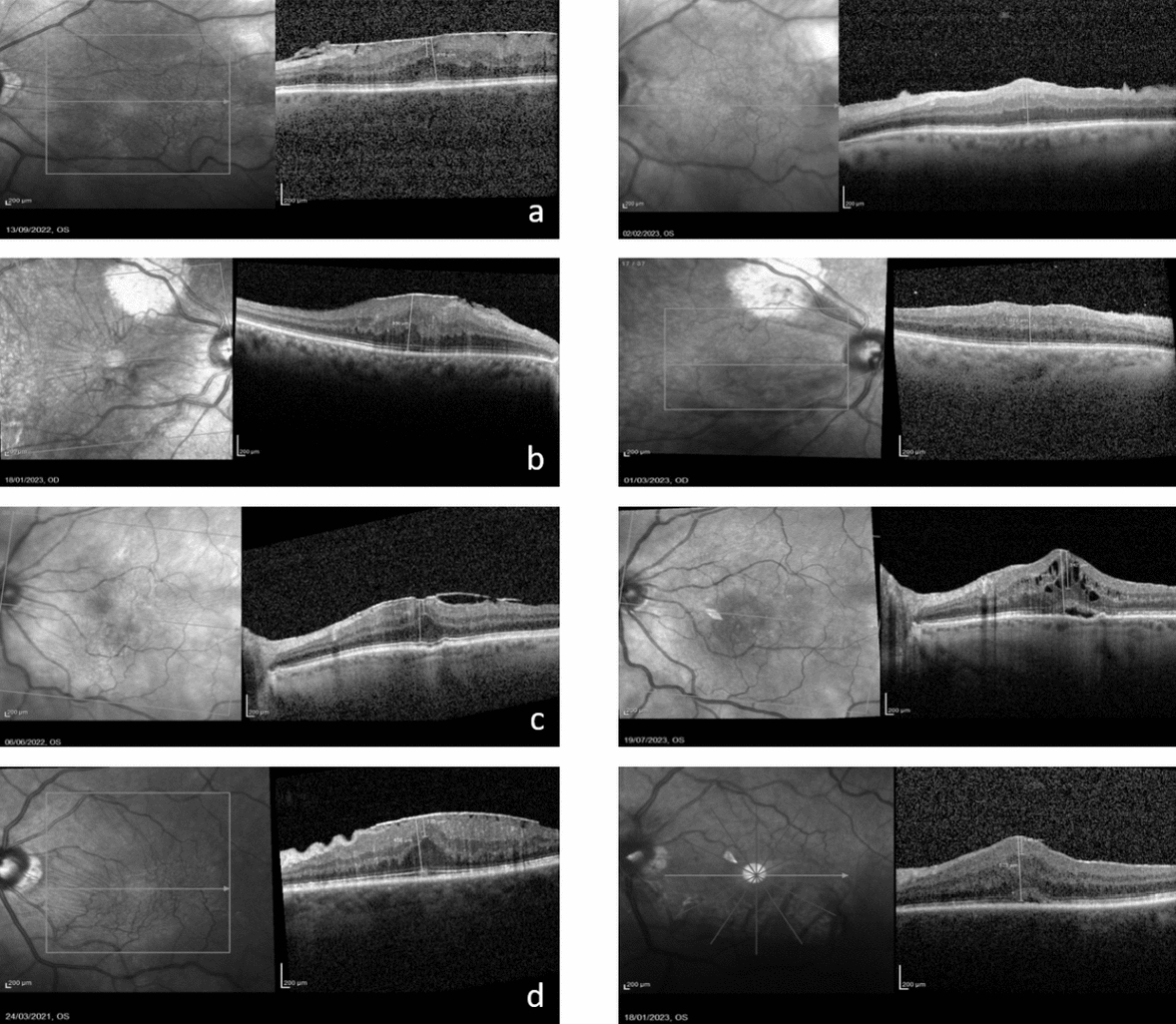
This figure shows the OCT images of 4 patients before (left) and after (right) vitrectomy with IVTA **a** 80 year old female patient with S-3 iERM in LE. CRT: 416 μm; EIFL-T: 174 μm; BCVA 20/70. After 5 months CRT was 401 μm, EIFL-T 262 µm, note the persistence of central foveal thickening; BCVA 20/33. **b** 74 year old female patient with S-3 iERM in her LE. CRT: 550 μm; EIFL-T: 306 μm; BCVA: 20/70. After vitrectomy with IVTA CRT decreased to 272 μm, EIFL-T 133 μm, slightly reappearance of the foveal depression; BCVA: 20/28. **c** 66 year old female patient with S-3 iERM in LE. BCVA: 20/49, CRT: 377 μm, EIFL-T: 137 μm. After 12 months CRT: 502 μm, EIFL-T: 307 μm, chronic CME; BCVA 20/49. **d** 66 year old male patient with S-3 iERM in LE. BCVA: 20/66, CRT: 558 μm, EIFL-T: 175 μm. After 12 months CRT: 523 μm, EIFL-T: 145 μm, note the persistence of central foveal thickening; BCVA 20/33. *IVTA* intravitreal injection of triamcinolone acetonide, *S* Govetto Stage, *iERM* idiopathic epiretinal membrane, *LE/RE* left/right eye, *CRT* central retinal thickness, *EIFL-T* ectopic inner foveal layer thickness, *BCVA* best corrected visual acuity in Snellen equivalent, *CME* cystoid macular edema

At the end of follow-up, in some cases the EIFL disappeared with recovery of the foveal depression. This happened in 10 patients in G-2 (43%), but only in 4 patients in G-1 (16%). (Figs. 2, 3).

Four patients in G-1 (16%) developed signs of cystoid macular edema (CME) occurring on average 25 ± 15 days after surgery and were treated with Fluocinolone 0.04% eye drops 4 times daily for one month. In two cases that did not respond to topical therapy, a second SChI-TA was performed with resolution of CME in agreement with the patients and after discussion of potential adverse events.

None of the G-2 patients developed CME within three months of surgery. One patient (4.3%) developed CME approximately 100 days after surgery. This patient was immediately treated with a second SChI TA, which permanently resolved the CME as he needed to renew his driving licence. We found no significant changes in IOP within the groups (RMANOVA p > 0.05) and between groups (t-test > 0.05) at the end of the study.

An IOP increase of 10 mmHg was reported in 1 patient in G-1 and in 1 patient in G-2 one month after the procedure; topical therapy was sufficient to reduce IOP to less than 20 mmHg in both cases. At the end of follow-up, no patients needed to continue hypotonizing therapy. No serious intraoperative and postoperative complications were recorded during the follow-up.

## Discussion

The main finding from this study is that the use of SChI-TA after surgery is a repeatable and safe procedure that provides significant recovery of BCVA and reduction of macular CRT and EIFL-T. In addition, TA was more effective when administered suprachoroidally than intravitreally.

TA is a synthetic lipophilic intermediate-acting corticosteroid. Because of its low solubility in aqueous solution, it can be used in sustained-release crystalline form, making it suitable for local depot injection. TA has been known to reduce inflammation and suppress cell proliferation increasing the production of basic fibroblast growth factor (bFGF) and decreasing the production of transforming growth factor beta (TGF-β) by human fibroblasts [13].

Until recently, the only method to deliver high doses of TA to the retina after vitrectomy was by intravitreal injection. However, IVTA can be associated with risks as direct contact and toxicity to the sensory retina, risks of infection, and of increased IOP. Studies in vitrectomized eyes have reported that the average elimination half-life of a 4 mg IVTA is 2–3 days, so the drug remains in the vitreous for less than 10 days [7, 14].

After vitrectomy, the anti-inflammatory effect of IVTA wanes rather quickly and is sometimes followed by a rebound effect of macular edema [15]. The duration of IVTA can reach several weeks raising the dosage to 20 mg, but with the risk of major complications such as glaucoma [8].

The efficacy of intravitreal corticosteroids after vitrectomy for iERM can be increased by using a sustained release dexamethasone intravitreal implant (Ozurdex^®^ 0.7 mg-Allergan-Irvine-CA) [16].

The effect of Ozurdex^®^ in a vitrectomized eye lasts for several months, and its use after vitrectomy for iERM has shown positive results with faster improvement in visual acuity and faster resolution of macular edema compared to untreated patients [17].

However, Ozurdex^®^ is not always routinely available, is expensive, and injection into a vitrectomized eye may cause some complications. There is a possibility of retinal injury due to impact; mobility of the implant in the vitreous chamber may cause floaters, migration into the anterior chamber, corneal decompensation, etc.

A new route of drug delivery into the eye has recently been discovered: the injection into the SChS, a virtual space located between the sclera and the choroid [18]. TA is one of the best preparations that can be injected into the SChS due to its lower solubility and prolonged release [19–21].

Animal and human studies have shown that TA administered suprachoroidally remains in the ocular tissues for at least 3 months, longer than the intravitreal route, and is unaffected by whether the eye has undergone vitrectomy. SChI-TA induces significant concentrations of steroid in the retina, choroid and sclera, with small amounts reaching the lens and anterior chamber.

Numerous trials have confirmed the clinical validity of this procedure in the treatment of macular edema caused by uveitis, diabetes or retinal vascular accidents [22–24].

Idiopathic ERM is a macular disorder characterized by fibrocellular proliferation and contraction on the central retina. Müller cells are the major cellular components of iERM. Müller cells become ‘reactive’ in response to virtually every pathological stimulation of the retina. This reaction is called Müller cells gliosis or, precisely in iERM: glial to mesenchymal transition (GMT) that is a transdifferentiation process characterized by the downregulation of Müller cells glial markers, paralleled by the upregulation of pro-fibrotic myofibroblast markers [25].

Various cytokines and growth factors act as modulators by triggering Müller cells swelling, transdifferentiation, proliferation, collagen production and contraction. TGF β appears to be the major cytokine driving GMT in iERM [26].

Recently, Govetto et al. introduced the new SD-OCT-based ERM staging scheme based on the presence or absence of EIFL. EIFL is defined as the presence of a continuous hyporeflective (extension of the inner nuclear layer) or hyperreflective (extension of the inner plexiform layer) band across the fovea [11].

Gliosis and straightening of the perifoveal Müller cells due to the anteroposterior and centripetal traction forces of the ERM are thought to be responsible for the progressive displacement of the inner nuclear and plexiform layers through the foveal centre, creating the EIFL.

The classification of ERM is divided into four stages: in S-3 the retinal layers are clearly visible under EIFL, in S-4 the retinal layers are not clearly distinguishable. The presence of EIFL is a sign of increased Müller cell activation and GMT and an independent predictor of worse postoperative visual acuity [27]. Accordingly, we chose to treat patients with S-3 and S-4 iERM with TA. Surgical removal of the iERM eliminates the mechanical stimulus that maintains and worsens Müller cell GMT over time, leading to slow and often incomplete recovery of macular structure and improvement in BCVA.

However, in the early postoperative period surgery cannot modify the concentration of cytokines within the retinal parenchyma, which maintains the reactivity of Müller cells. Pre-existing pathologic edema due to ERM may be slow to disappear, persist, or even worsen following the trauma of mechanical peeling of the membrane.

Although ILM peeling in iERM surgery is controversial [28], ILM peeling may be considered a recommended procedure in the treatment of advanced iERM with severe macular distortion, as it reduces the risk of re-intervention.

In our experience, if ILM peeling is not performed in the advanced stages of iERM, recurrence of macular pucker is quite common [29], so we always perform this procedure, even though it may be one of the causes of the onset of CME in the postoperative period. In fact, surgical trauma to the retinal tissue during ILM peeling causes postoperative macular changes such as thickening, thinning or dimpling [30]. Disruption of adhering Müller cell end-feet during ILM peeling may cause a transient increase in local production of inflammatory cytokines that in some cases can cause CME [31].

The rationale for using TA that inhibits cell proliferation and inflammation is to both to inhibit the production of local modulators, including TGFβ, responsible of retinal gliosis, and to reduce the incidence of CME. IVTA used intraoperatively during vitrectomy for iERM removal has shown mixed results. Some authors demonstrated that intraoperative IVTA had a beneficial effect on the rapidity of visual recovery due to faster fluid absorption, prevention of postoperative inflammation and neuroprotective action on the photoreceptors [2–4], while others have not found differences in final results between treated and untreated patients [5, 6].The conflicting results between authors may be explained by the rapid clearance of TA in vitrectomized eyes [7].

In this study, 4 mg SChI-TA was advantageous compared to 4 mg IVTA because it offered a longer duration of action, possibly corresponding to a higher intraocular TA level. This is consistent with studies using Ozurdex^®^ during iERM surgery [16].

At the end of the study, G-1 had a mean reduction in CRT and EIFL-T of − 131 μm (− 26.4%) and − 104 μm (− 46.6%), respectively, while G-2 had a significantly greater reduction in both parameters: − 222 μm (− 39.1%) and − 200 μm (− 68%). (Table 3).

**Fig. 4:**
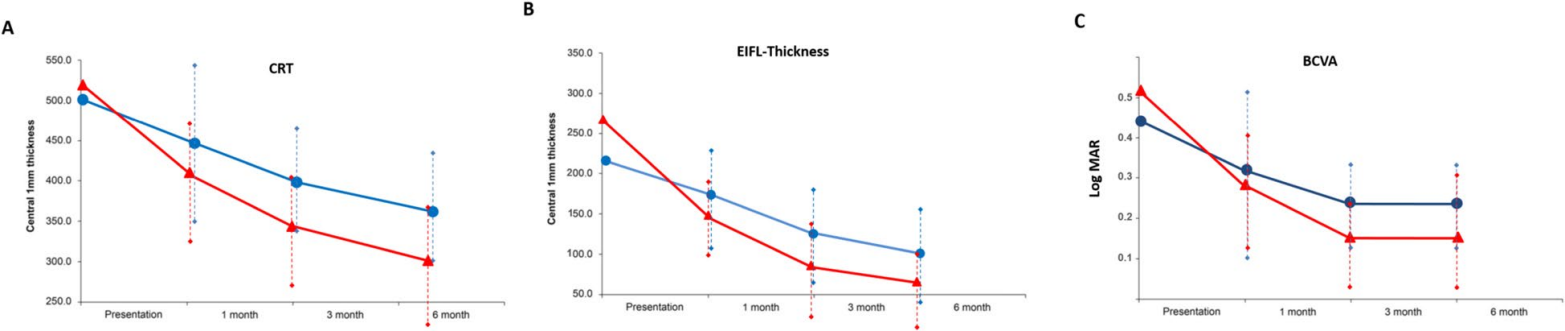
Graphs **A** Central retinal thickness (CRT), **B** Ectopic inner foveal layer thickness (EIFL-T), and **C**—Best corrected visual acuity (BCVA) in patients who underwent vitrectomy and intravitreal injection of triamcinolone acetonide (IVTA, blue dots) or suprachoroidal injection of triamcinolone acetonide (SCHi-TA, red triangles), mean ± SD

This correlated with greater increase in BCVA in patients undergoing SCh-TA. (Fig. 4).

Our results support that changes in macular thickness after iERM peeling have a significant inflammatory component; therefore, prolonged anti-inflammatory medication is useful. EIFL-T was significantly affected by the sustained release of TA: at the end of the study, the EIFL was no longer measurable in 10 cases of G-2 (43%) and 4 cases of G-1 (16%). Prolonged inhibition of local cytokine and TGF-β production, which results in immune activation of Müller cells, was able to partially or completely regress the gliotic responses of Müller cells.

We were unable to determine with certainty how long the iERM had been present in most cases. It is possible that in cases where the iERM has recently formed, the reactive inflammatory component predominates over the structural component, and therefore surgery added to TA may be more effective in restoring macular integrity in the early stages.

Further studies should be conducted to determine the benefits of early surgery for iERM compared to watchful waiting and late surgery.

According to the literature, postoperative CME occurs in 13–47% of patients after ERM/ILM peeling and is a serious event responsible for the delay in visual recovery in these patients [31, 32].

Within 3 months of surgery, 16% of G-1 patients and no G-2 patients developed signs of CME. However, one patient in G-2 (4.3%) developed CME after 3 months. This indicates that the TA was washed out from the suprachoroidal space; that is a warning that the rebound effect of CME is possible even after SChI-TA. We remind that all the patients of the study were pseudophakic. Regarding IOP, the prevalence of ocular hypertension after SChI-TA was comparable to that after IVTA (~ 4%), transient and controllable with topical therapy.

A major limitation of the current study was the small sample size and relatively short follow-up. However, postoperative changes in CRT, EIFL-T, IOP and incidence of CME occur mainly in the first 6 months after iERM removal, whereas changes after 6 months were reported to be minimal. Inaccuracy of manual measurement of CRT and EIFL-T was another confounding factor in this study. However, the measurement was performed in a masked fashion by three different authors, and the reduction in both parameters was undoubtedly greater in G-2.

In conclusion, SChI-TA promoted a greater reduction of CRT and EIFL-T than IVTA after vitreoretinal surgery for severe iERM. With SChI-TA, 43% of patients (compared to 16% with IVTA) had a recovery of foveal depression. This was associated with a greater improvement in BCVA. Patients treated with SChI-TA had fewer cases of postoperative CME than those treated with IVTA. However, the rebound effect of CME may also occur after SChI-TA. Finally, SChI-TA did not increase the risk of ocular hypertension compared to IVTA.

These data may support the routine use of SChI-TA in combination with standard vitrectomy and peeling for the treatment of advanced stage iERMs.

## Data Availability

The datasets used and/or analysed during the current study are available from the corresponding author on reasonable request.

## Abbreviations

iERM: Sidiopathic epiretinal membranes
PPV: Pars-plana vitrectomy
ILM: Inner limiting membrane
IVTA: Intravitreal injection of triamcinolone acetonide
BCVA: Best corrected visual acuity
CRT: Central retinal thickness
SChI-TA: Suprachoroidal injection of triamcinolone acetonide
SchS: Suprachoroidal space
OCT: Optical coherence tomography
IOP: Intraocular pressure
SE: Snellen Equivalent
EIFL-T: Ectopic inner foveal layer thickness
G: Gauge
G-1: Group-1
G-2: Group-2
SD: Standard deviation
LE: Left eye
CME: Cystoid macular edema
bFGF: Basic fibroblast growth factor
TGF-β: Transforming growth factor beta
GMT: Glial to mesenchymal transition
